# Conspecific and heterospecific pheromones stimulate dispersal of entomopathogenic nematodes during quiescence

**DOI:** 10.1038/s41598-020-62817-y

**Published:** 2020-03-31

**Authors:** Fatma Kaplan, Abigail Perret-Gentil, Julie Giurintano, Glen Stevens, Hilal Erdogan, Karl C. Schiller, Amaleah Mirti, Edith Sampson, Cedric Torres, Jiayi Sun, Edwin E. Lewis, David Shapiro-Ilan

**Affiliations:** 1 Pheronym, Inc., Davis, CA 95618 USA; 2 University of Idaho, Department of Entomology, Plant pathology and Nematology, Moscow, ID 83844 USA; 3 USDA-ARS, Southeastern Fruit and Tree Nut Research Laboratory, Byron, GA 31008 USA; 4 Faculty of Agriculture, Department of Biosystems Engineering, Bursa Uludağ University, Bursa, 16059 Turkey; 5 Present Address: GRACE Market Place, Gainesville, FL 32609 USA; 6 Present Address: Lewis Katz School of Medicine, Philadelphia, PA 19140 USA; 7 Present Address: University of Florida, Gainesville, FL 32610 USA; 8 Present Address: Mérieux NutriSciences, Gainesville, FL 32641 USA; 9 Present Address: Captozyme, LLC., Gainesville, FL 32653 USA

**Keywords:** Chemical ecology, Behavioural ecology, Chemical biology, Ecology

## Abstract

Ascaroside pheromones stimulate dispersal, a key nematode behavior to find a new food source. Ascarosides produced by entomopathogenic nematodes (EPNs) drive infective juvenile (IJ) emergence from consumed cadavers and dispersal in soil. Without ascarosides from host cadavers, *Steinernema feltiae* (EPN) reduce dispersal substantially. To determine whether other *Steinernema* spp. exhibit the same behavior, we compared *S. feltiae* and *S. carpocapsae* IJs without host cadaver pheromones. Unlike *S. feltiae*, *S. carpocapsae* IJs continued to disperse. However, *S. carpocapsae* IJs exhibited a temperature-dependent quiescent period. The IJ quiescent period increased at ≤20 °C but did not appear at ≥25 °C. Consistent with this, *S. carpocapsae* IJ quiescence increased from 30 min to 24 h at ≤20 °C over 60 days. The quiescent period was overcome by dispersal pheromone extracts of their own, other *Steinernema* spp. and *Heterorhabditis* spp. Furthermore, *S. carpocapsae* IJ ambush foraging associated behaviors (tail standing, waving, and jumping) were unaffected by the absence or presence of host cadaver pheromones. For *S. feltiae*, IJ dispersal declined at all temperatures tested. Understanding the interaction between foraging strategies and pheromone signals will help uncover molecular mechanisms of host seeking, pathogenicity and practical applications to improve the EPN’s efficacy as biocontrol agents.

## Introduction

Dispersing and host-finding are important behaviors for parasites’ success; finding a new host involves emergence from the host, dispersal, and foraging for a new host. A combination of intrinsic chemical drivers and extrinsic cues associated with potential hosts drives dispersal, searching behaviors and infection decisions. Local environmental conditions also affect parasites’ success. For example, for soil-associated parasites these environmental factors include soil type, soil moisture, salinity, and temperature, among others.

Entomopathogenic nematodes (EPN) in the genera *Heterorhabditis* and *Steinernema* are insect parasites used as model organisms to study the biology of parasites^1,2^. Since they kill insects, they also have commercial applications for controlling insect pests as biocontrol agents^3–5^. Once EPNs consume an insect host a specialized non-developmental life stage, called the infective juvenile (IJ), emerges from the spent host and disperses to search for a new host. The IJs carry tens to hundreds of symbiotic bacteria cells (*Xenorhabdus* spp. for *Steinernema* spp. nematodes and *Photorhabdus* spp. for *Heterorhabditis* spp. nematodes). Once a host has been infected by multiple IJs, the nematodes resume development, release their symbiotic bacteria, feed on the bacteria, and one to three generations develop within a single host over a 10 to 22-day period. When nutritional quality declines and waste products increase, IJs once again develop and emerge from the host. The EPN IJ host finding process includes behavioral stages: emergence (exodus) from the consumed insect cadaver, dispersal through the soil, and foraging for new hosts.

Biotic and abiotic factors influence IJ behaviors. These factors may act inside and outside the host cadaver. EPNs survive within water films in interstitial spaces in soil, where factors such as moisture and soil type affect survival and dispersal^6–12^. Furthermore, EPNs outside the cadaver respond directionally to a variety of stimuli such as CO_2_^13,14^, vibration^15^, temperature^16^, nitrogen-based compounds^17^, electromagnetic stimuli^18,19^, volatile cues from infected and uninfected insect hosts^14,20,21^ and pheromones^22,23^. Interspecific variation in foraging strategy also influences behavior^4,11,14,24^. All species move through the soil matrix, but ambush specialists (e.g., *S. carpocapsae, S. scapterisci* and *S. siamkayai)* exhibit tail-standing, waving and jumping behaviors that define the strategy^25,26^.

**Figure 1:**
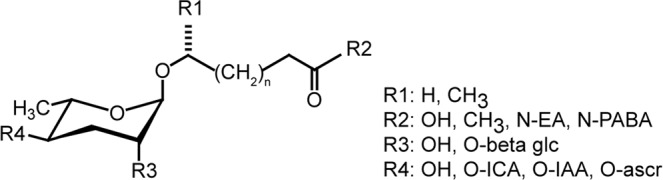
General structure of ascaroside pheromones. N-: N linked; O-: O linked; EA: ethanolamine; PABA: para amino benzoic acid; ICA: indole carboxylic acid; IAA: indole acetic acid; beta glc: beta glucose; ascr: ascaroside.

Pheromones affect nematode behavior and development broadly throughout the phylum. Nematode pheromones, called ascarosides, are produced by many nematode species^22,27–30^. Ascarosides are a class of compounds (Fig. 1) composed of a central ascarylose sugar with a variable lipid side chain, and both the lipid chain and ascarylose sugar can have modifications^22,27–29,31–34^. In free-living nematodes, individual ascarosides and mixtures regulate mating, aggregation, attraction, repulsion and dispersal behaviors^22,30,35,36^.

**Figure 2:**
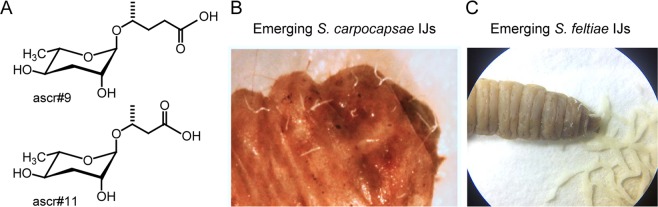
Pheromones and their role in EPN emergence. (**A**) Structure of ascaroside pheromones found in EPN infected insect host cadavers. Asrc#9 and ascr#11 are identified as part of the pheromone mix responsible for causing the exit of IJs from the consumed insect host cadavers or exodus pheromone signal (**B**) Consumed insect host cadaver with emerging *S. carpocapsae* IJs. (**C**) Consumed insect host cadaver with dispersing *S. feltiae* IJs. Image courtesy of Pheronym.

Ascarosides play a key role in EPN development and behaviors^22,29,37–41^ including preventing the recovery of IJs (analogous to the dauer stage in *C. elegans*), emergence from host cadavers after food depletion, and dispersal behavior after IJs emerge from the host^22,29^. While their significance is known, the specific compounds produced within an EPN system and their effects on behavior vary. For example, ascr#9 and ascr#11 (Fig. 2A), from consumed insect host cadavers (Fig. 2B,C), play a role in *S. feltiae* IJ dispersal as part of a pheromone mixture^22^. Ascr#9 and ascr#11 are also structural analogs and are interchangeable in the dispersal pheromone mixture^22^. Furthermore, ascr#9 is found in host cadavers of other *Steinernema* spp.^22^ (*S. feltiae*, *S. carpocapsae*, *S. diaprepesi*, *S. riobrave*) and *Heterorhabditis* spp.^22^ (*H. bacteriophora*, *H. floridensis*, *H. zealandica*), suggesting that behavioral responses to EPN pheromones in spent host cadavers may be broadly conserved.

Understanding pheromone effects on dispersal has significant implications to translational and applied sciences because EPNs are commercially available biological control agents and also used as model organisms to study the biology of parasites^1–5^. Consistent with Kaplan *et al*.^22^ findings, ascaroside-containing extracts from host cadavers increased *S. feltiae* and *S. carpocapsae* IJ dispersal^23^ by a factor of 3 and led to increased insect host encounter 35 cm below the application site in soil columns. Furthermore, pheromone extract treated IJs have 28–78% better biocontrol efficacy against pecan weevil and black soldier fly larvae than that of non-treated IJs in greenhouse trials^23^.

Exposure to pheromone extracts improves IJ efficacy^23^, but we don’t know whether the behavioral patterns exhibited by the two species in the absence of pheromones are the same. The differences in foraging behaviors and associated hierarchical cues required to trigger infection decisions^42^ may result in different responses to pheromone extracts. We hypothesized that if dispersal is stimulated by ascarosides throughout the Nematoda, behavioral changes in *S. carpocapsae* in response to conspecific pheromone extracts (and their absence) would mirror those of *S. feltiae*, despite fundamental differences in their foraging behaviors. We assessed temporal patterns in dispersal behavior and thermal controls on dispersal behavior using a common assay (with or without pheromone under different conditions) for *S. feltiae* and *S. carpocapsae*. Marked differences between the two species led to follow-on experiments focused on *S. carpocapsae*. These included assessing ambusher-associated behaviors such as tail standing, waving, and jumping^26^. Finally, we assessed the dispersal response of *S. carpocapsae* to pheromone extracts from a range of EPN species that varied in their foraging behaviors and their phylogenetic relatedness. We discuss these results in the context of emergence, dispersal and foraging behaviors, thereby providing a framework to develop further hypotheses regarding the role of pheromones in this system.

## Results

### *S. carpocapsae* and *S. feltiae* respond differently to the absence of pheromones from spent host cadavers

**Figure 3:**
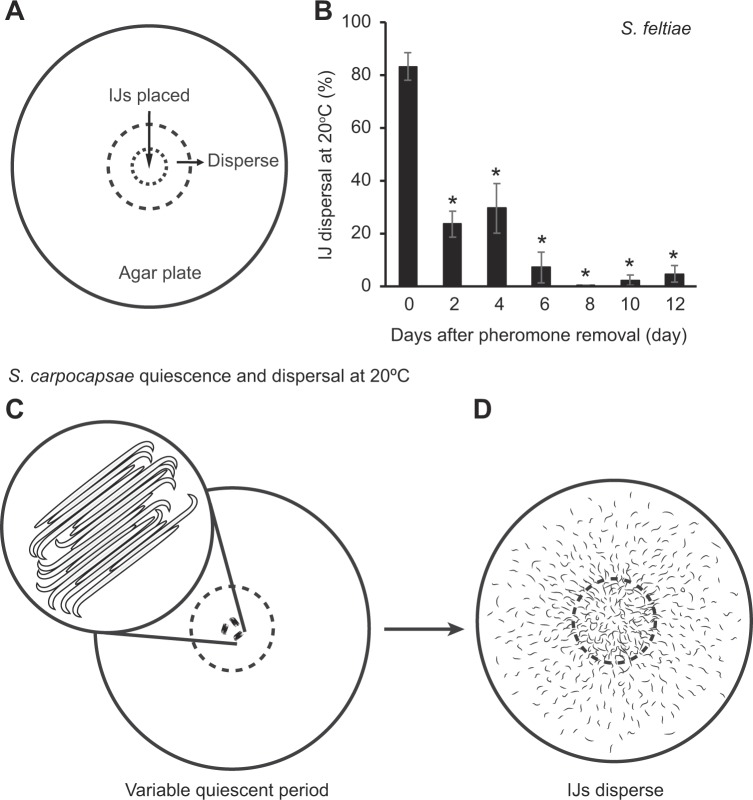
*Steinernema feltiae* and *Steinernema carpocapsae* IJs dispersal in the absence of pheromones from insect host cadavers. (**A**) Dispersal assay. IJs are placed in the center of a 6 cm diameter agar plate. The dashed lines represent a 1.3 cm diameter circle that marks the dispersal boundary. IJs that move outside the boundary are considered dispersed. Quiescent period is the time it takes IJs to resume normal activity and move toward the dashed lines. (**B**) *S. feltiae* IJ dispersal. The mean ± s.e.m. of 3 replications is presented. *Indicates statistically significant difference from Day 0 among means. Linear regression analysis. R^2^ = 0.93. (**C**) *S. carpocapsae* IJ quiescence. It is varaible with time and temperature. (**D**) *S. carpocapsae* IJ dispersal after quiescence.

To determine whether *S. feltiae* and *S. carpocapsae* IJs exhibit similar temporal patterns in dispersal behavior when they are stored without pheromones from consumed host cadavers, we followed methods described for the dispersal assay shown in Fig. 3A. Initial comparisons of dispersal between *S. feltiae* and *S. carpocapsae* were conducted at 20 °C in Fig. 3. As expected, *S. feltiae* dispersal declined significantly over time (Fig. 3B, F_6,14_ = 32 and P < 0.00001, Supplemental Table S2, F_7__,__24_ = 15.56, and P < 0.00001). Within 2 days of washing the IJs from residual pheromones, dispersal rates declined to between 20 and 30%, and by day 6, dispersal ranged from 0 to 20% (Fig. 3B, Supplemental Table S2, statistics for Supplemental Tables S1 and S3).

To compare *S. carpocapsae* dispersal we adapted the assay from *S. feltiae* and we noticed that *S. carpocapsae* IJs had a quiescent period preceding dispersal (Fig. 3C). The quiescent period is the duration IJs remain motionless after application, after the water was absorbed and IJs were therefore free to move. First we focused on *S. carpocapsae* IJ dispersal (Fig. 3D) which never showed the decline in dispersal that we observed in *S. feltiae*. This was surprising, considering the reports that both *S. carpocapsae* and *S. feltiae* IJs increased dispersal in response to pheromone extracts in sandy soil, but only *S. feltiae* IJs reduced dispersal in the absence of pheromones in Fig. 3B. Then we hypothesized that the quiescent period (Fig. 3C) may be affected by the absence of dispersal pheromone signals and measured the quiescent period.

### Quiescent period and dispersal are affected by temperature and pheromones during storage

**Figure 4:**
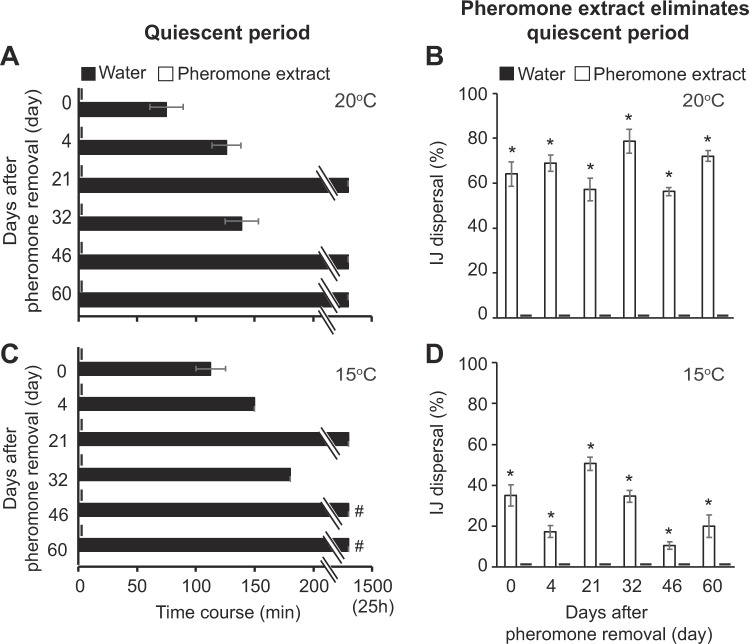
Time course for *Steinernema carpocapsae* IJ population quiescent period and pheromone stimulated dispersal. (**A**) The IJ quiescence time at 20 °C in the presence and absence of pheromone extracts. (**B**) Pheromone extracts stimulated percent IJ dispersal in the first 30 min whereas untreated IJs are quiescent at 20 °C. (**C**) The IJ quiescent period at 15 °C in the presence and absence of pheromone extracts. (**D**) Pheromone extracts stimulated IJ dispersal in the first 30 min when untreated IJs were quiescent at 15 °C. The mean ± s.e.m. of 4 replications is presented. ^#^Experiment stopped after 24 h. *Significantly different compared to control.

We investigated quiescent period and dispersal patterns during storage for both species because many commercial formulations of EPNs are stored for up to 60 days (2 months) before use and findings have practical applications for manufacturers and farmers. For *S. carpocapsae* IJs, we conducted time course experiments up to 60 days and determined quiescent period duration and dispersal at four temperatures. There was no consistent quiescent period observed at 25 and 30 °C, though IJs on some plates (12.5%) exhibited quiescence after 21 days; due to this general lack of quiescence, we did not test whether pheromone extract would stimulate IJ dispersal at 25 and 30 °C. At 20 °C and 15 °C, IJs on all plates exhibited quiescence, and the quiescent period increased during storage (Fig. 4A,C, Supplemental Tables S4 and S5) in the absence of pheromone extracts from spent host cadavers. At 15 °C, at days 46 and 60, the quiescence exceeded 24 h (Fig. 4C) so we stopped the experiments. *S. carpocapsae* IJs treated with pheromone extracts did not have a quiescent period at 20 °C or 15 °C (Fig. 4A,C, Supplemental Table S5) and were stimulated to disperse (Fig. 4B,D, Supplemental Table S5).

**Figure 5:**
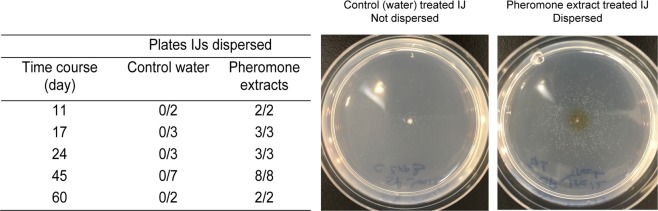
*Steinernema feltiae* IJ population dispersal in the absence and presence of pheromone extracts from consumed host cadavers. *Day 60 collection method was different where we used a pre-post research design. Replications (n) are presented in the figure. Image courtesy of Pheronym.

Next, we quantified pheromone extract-stimulated dispersal by allowing IJs to disperse for 30 min (Fig. 4B,D, Supplemental Table S5). Across the 60-day assay at 20 °C, between 56% and 78% of *S. carpocapsae* IJs treated with pheromone extracts dispersed, while untreated IJs remained quiescent (F_2,45_ = 455 and P = 2.2*10^−16^) (Fig. 4B and Supplemental Table S6). Similarly, at 15 °C, between 11% and 51% of pheromone extract treated IJs dispersed, while the water treated controls remained quiescent (F_2,45_ = 44.31 and P = 2.3*10^−11^) (Fig. 5D and Supplemental Table S7). We repeated the first 30 days and again pheromone extract from consumed host cadavers stimulated the dispersal during the quiescent period while the water treated controls remained quiescent at both 20 and 15 °C treatments (Supplemental Table S5). These results suggested that exposure to pheromone extracts overcomes the quiescent period and stimulates dispersal at 20 °C or lower.

After the quiescent period, we allowed IJs to disperse for 30 min. Dispersing IJ numbers for *S. carpocapsae* fluctuated at all temperatures (Supplemental Fig. S1 and Table S5). However, overall dispersal rate was reduced at 15 °C compared to 25 and 30 °C in the absence of pheromones (Supplemental Fig. S1A,B, Table S5). Pheromone extracts treatment had a positive effect on IJ dispersal at 20 and 15 °C. For 25 and 30 °C, there was no quiescent period when IJs were allowed to disperse 30 min. At 20 and 15 °C, IJ dispersal was quantified after the quiescent period (30 min dispersal) suggesting that quiescence can also be overcome by temperatures above 20 °C and/or pheromone extracts.

We also monitored *S. feltiae* IJ dispersal during storage. A sixty-day time course (Fig. 5) for *S. feltiae* IJ dispersal was conducted at 20 °C because temperature range did not seem to affect IJ dispersal temporal pattern (Supplemental Fig. S2) other than reducing the overall dispersal rate. Consistent with previous tests, on day 11 and thereafter, *S. feltiae* IJs stayed at the introduction site and rarely, if ever, moved over the 30-minute assay (Fig. 5). Treatment of *S. feltiae* IJs with pheromone extracts caused them to disperse immediately (Fig. 5).

### Pheromone extract affects *S. carpocapsae* dispersal but not tail standing, waving or jumping

*S. carpocapsae* and *S. feltiae* have different foraging strategies, ambush and intermediate, respectively. Since *S. carpocapsae* responded differently from *S. feltiae* IJs, we investigated other behaviors associated with the ambushing foraging strategy, including tail standing, waving and jumping. Plain water agar assays cannot test behavior associated with ambushing because the water surface tension prevents IJs from tail standing or waving. Therefore, we added sand to the surface of agar plates to allow the IJs to tail stand, wave, and jump.

We first determined whether there was a difference in IJ crawling in plates with and without sand, as opposed to demonstrating ambushing behaviors. The addition of sand to the agar arenas significantly reduced crawling (F_1,19_ = 60.7, P < 0.0001). In plates without sand, 99.9% of the assessed nematodes were found to be crawling; in plates with sand, 68.4% of the assessed nematodes were crawling. Furthermore, there was no effect of pheromone extracts on crawling (F_1,19_ = 0.001, P = 0.97) and no interaction between sand and pheromone extract treatments (F_1,19_ = 0.001, P = 0.97).

**Figure 6:**
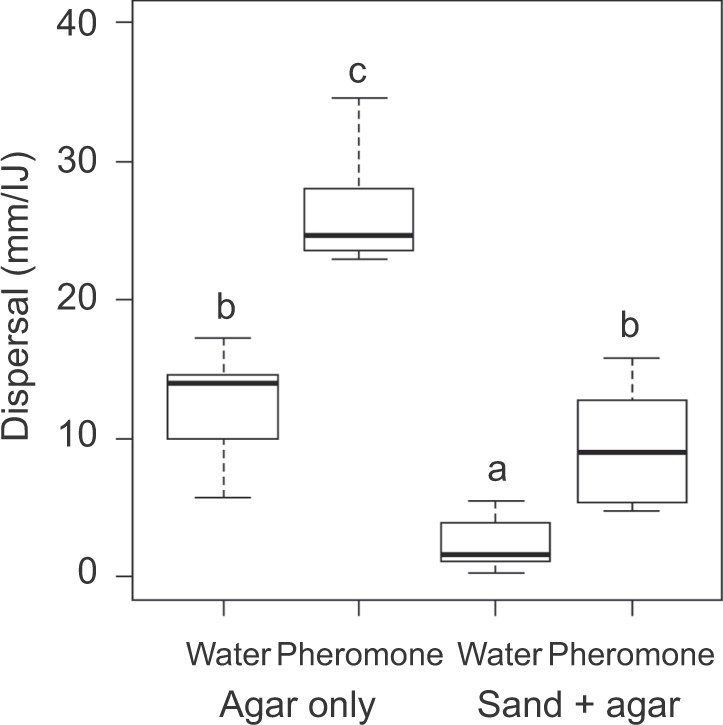
Dispersal after 30 min for *Steinernema carpocapsae* in response to exposure to pheromone extracts (treatment, pheromone) or water (control) at 22 °C and the addition of sand particles to an agar arena. Boxplots show median (dark line), first and third quartiles, and minimum and maximum values. Different lowercase letters indicate significant differences among treatments according to Tukey’s HSD (α = 0.05).

In dispersal plate assays, we determined how far IJs moved from the placement site. The addition of sand decreased dispersal rates (F_1,20_ = 73.0, P < 0.0001) and treatment with pheromone extracts increased dispersal rates (F_1,20_ = 43.2, P < 0.0001). Furthermore, the proportional effect of the pheromone extracts on dispersal was stronger in dishes with sand. On plain agar, dispersal rates were greater than on agar + sand plates, and exposure to pheromone extracts nearly doubled average dispersal rates. Treating IJs with pheromone extracts on agar plates with sand quadrupled dispersal rates (sand*pheromone interaction, F_1,20_ = 4.5, P = 0.046, Fig. 6).

Adding sand to the arenas also increased the number of IJs found on the dish lid (i.e., nematodes that jumped, F_1,20_ = 9.5, P = 0.0059). However, the treatment with pheromone extracts did not affect jumping (F_1,20_ = 0.39, P = 0.54) and there was no interaction between sand and pheromone treatments (F_1,20_ = 0.337, P = 0.57).

### *S. carpocapsae* IJs respond to dispersal pheromones from other *Steinernema* spp. and *Heterorhabditis* spp. infected host cadavers

**Figure 7:**
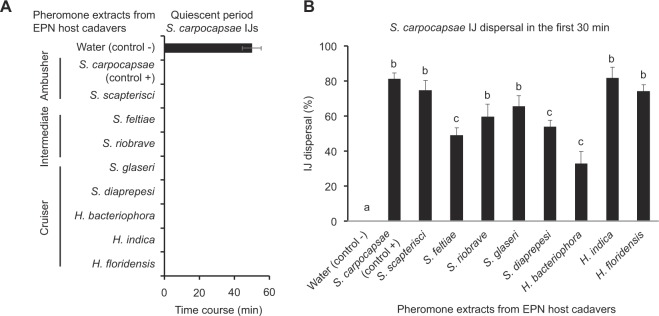
*Steinernema carpocapsae* IJ quiescent period and pheromone extract induced dispersal from EPN host cadavers with different foraging strategies. Assays conducted at 21.5 ± 1 °C. (**A**) Quiescent period in the absence (water control) and presence of pheromone extracts. The mean ± s.e.m. of 9 replications from two assays is presented. (**B**) Pheromone extract stimulated dispersal within the first 30 min. The mean ± s.e.m. of 5 replications is presented. Different lowercase letters indicate significant differences among treatments according to Tukey’s HSD (α = 0.05).

Spent host cadavers infected by seven different EPN species have at least one component (ascr#9) of the dispersal pheromone mixture in common suggesting that EPNs may recognize each other’s dispersal signals from consumed host cadavers. To determine whether *S. carpocapsae* IJs recognize dispersal signals from EPNs with different levels of phylogenetic relatedness (congeners vs. more distantly related species) and different foraging strategies, we tested dispersal pheromone extracts from 8 EPN species on the duration of IJ quiescent periods. The foraging strategies included ambushers (*S. carpocapsae* [positive control] and *S. scapterisci*), cruise foragers (*S. glaseri*, *S. diaprepesi*, *H. bacteriophora*, *H. indica* and *H. floridensis*) and intermediates (*S. feltiae* and *S. riobrave*). *S. carpocapsae* IJ quiescence period is shortened by exposure to pheromone extracts from host cadavers of EPNs; *Steinernema* spp. and *Heterorhabditis* spp. (Fig. 7A). Water-only treated *S. carpocapsae* IJs showed an average of 50 min quiescent period whereas pheromone extract treated IJs showed no quiescent period (Fig. 7A). Next, we allowed pheromone extract treated IJs to disperse for 30 min and quantified dispersal (Fig. 7B). Each of the pheromone extract treatments promoted IJ dispersal more than the water-only control (F_6,14_ = 8.755, P < 0.001), although there was some variability in the strength of the effect (Fig. 7B and Supplemental Table S8A,B and C). Our results suggest that cadaver-derived pheromone extracts from different EPN species have enough in common that they promote IJ dispersal across species.

## Conclusions and discussion

*S. feltiae* IJ dispersal was reduced so much during 60-day storage in water that it essentially stopped in the absence of pheromones from consumed host cadavers. This was consistent with the findings of previous studies^22^. Recently, Oliveira-Hofman *et al*.^23^ reported that when *S. feltiae* and *S. carpocapsae* IJs were treated with dispersal pheromone extracts, both species increased dispersal in 35 cm soil columns baited with *Tenebrio molitor*. Since *S. feltiae* IJs essentially stop dispersing during storage in the absence of pheromones from host cadavers, we hypothesized that both *S. feltiae* and *S. carpocapsae* IJs reduced their dispersal in the absence of pheromones. To our surprise, *S carpocapsae* IJ dispersal was not reduced like that of *S. feltiae*. Furthermore, IJ dispersal response to temperature in the absence of consumed host cadaver pheromones differed between species. Since *S. carpocapsae* and *S. feltiae* have different foraging strategies, perhaps the difference is due to foraging strategies.

*S. carpocapsae* IJ dispersal shows two distinct stages in the absence of pheromone signals from host cadavers: a quiescent period, which is the resting period before IJs resume motion, and a dispersal phase when they move away from the application site. Quiescence has been shown to result from exposure to root cap exudates^43,44^; this is a reversible state in which juvenile nematodes (including EPNs, *C. elegans*, and the plant parasitic nematode *Meloidogyne incognita*) become motionless and do not exhibit sinusoidal movement^44^. Exudate-induced quiescence has been defined as 100% lack of movement in all individuals in a population^43^; thus, what we observed in *S. carpocapsae* is a true quiescent period, as compared to what we saw for *S. feltiae*, which were low rates of movement and dispersal. Our results suggest that this quiescent period is influenced by both temperature and cadaver-derived pheromone extracts. At temperatures of 25 and 30 °C, no quiescence was observed, but at 20 and 15 °C, a quiescent period was observed. Quiescence seemed to be eliminated somewhere between 20 and 25 °C as we see no quiescence at 22 ± 1 °C in Fig. 6. In addition to assay temperature, there were substantial differences in the experimental protocol (depicted in Fig. 6) where quiescence was not observed. Why and how a temperature-related switch in quiescence occurs needs further study. Furthermore, the duration of quiescence at 20 and 15 °C increased from 50 min to 24 h across a two-month storage period. As expected from a pheromone induced behavior, IJ dispersal was stimulated by exposure to pheromone extracts at 20 and 15 °C during quiescence. Currently, we do not know whether the root cap exudates and dispersal pheromones target the same molecular pathways related to the quiescent period in *S. carpocapsae*.

**Figure 8:**
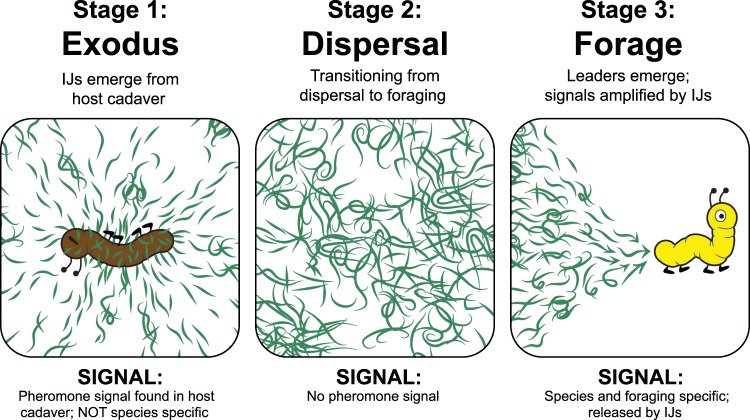
Model for ascaroside pheromones’ role in EPN emergence, dispersal and foraging. **Stage 1** is the exodus signal. It is expected to be recognized by all EPNs for emergence from host cadaver. **Stage 2** dispersal is the transition state from uncoordinated to coordinated movement. i.e. from randomly dispersing away from the cadaver to foraging. Stage 2 happens in the absence of pheromone signals from consumed host cadavers in the soil. **Stage 3** group foraging, IJs form leaders and followers. Pheromone signals are expected to be released by IJs and be species specific. Image courtesy of Pheronym.

To understand pheromones’ role in EPN emergence, dispersal, and foraging and how pheromones can be used to improve EPNs’ success as biocontrol organisms, we developed a model (Fig. 8). Based on prior literature, finding a new host includes distinct stages; emergence (exodus) from the consumed insect cadaver, dispersal, and foraging for new insects in the soil^11,12,22,25,38,42,45–47^. We defined these 3 stages so we can make hypotheses to elucidate pheromones’ role.

We hypothesize that **stage 1 (Exodus)**, includes exit from the cadaver and flight from the vicinity of the cadaver; in this stage pheromones signal IJs to emerge from the host cadaver due to overcrowding and lack of food. During this period nematodes infect hosts opportunistically. We hypothesize that during this stage, the pheromone signal is neither species-specific nor influenced by foraging strategies. In this study, *S. carpocapsae* IJs responded to pheromone extracts from consumed insect host cadavers of 8 different species of *Steinernema* and *Heterorhabditis* regardless of their foraging strategies. The literature and our current findings suggest that the pheromone mixtures have not diverged to the point where they are species specific^22,39,48^. However, the precise pheromone mix for each species remains to be discovered. We don’t know whether this mixture is composed of just ascaroside pheromones or includes non-ascaroside compounds.

In **stage 2 (Dispersal)**, if EPN IJs do not encounter a suitable host within a few days after emergence, the residual dispersal pheromone signal wanes as the nematodes move through water films in the soil. Subsequently, IJs are not exposed to cadaver-derived pheromones until they join another infection. We suggest that pheromone signals’ effect from the host cadaver wears off and internal signals take charge of movement as IJs transition to foraging behavior.

Currently we do not know whether pheromones are involved in transitioning to foraging behavior. In insects, lack of a juvenile hormone (JH) in the last larval stage triggers the metamorphosis to adult^49–51^. JH presence prevents the metamorphosis, meaning the larval stage is preserved and larvae continue molting^49–51^. If we apply the same concept from dispersal to foraging behavior, it may be that cadaver-derived pheromones prevent EPNs from organized foraging strategy, and that host cadaver signal must decline before EPN IJs can begin transition to foraging behavior. When *S. feltiae* IJs stop dispersing or *S. carpocapsae* IJs show quiescence, they stay as a group at the deployment spot on the agar plate in the absence of dispersal pheromone from host cadavers. This may be the start of the group or aggregative movement as reported by Ruan *et al*.^12^.

In **stage 3 (Group Foraging)**, IJs have transitioned to foraging. *C. elegans* is stimulated by ascaroside pheromones to aggregate^35^, and EPNs also move in groups^12^. We hypothesize that EPNs are driven to form groups by ascaroside aggregation pheromones. Consistent with the literature, Hartley *et al*.^39^ showed that as the ascaroside content increases during storage and overall nematode dispersal was reduced from 1 to 3 weeks. When the IJs are exposed to one week-storage medium associated with high dispersal, it stimulates the IJ dispersal as expected^39^. However, the 3-week storage medium with lower dispersal activity has a different ascaroside composition. Considering different compositions of ascarosides produce different activities, it is possible that the ascaroside composition in the 3-week storage medium has a different function such as forming groups or aggregation. We hypothesize that in the soil, pheromone signals are released by the IJs in response to cues from the environment; e.g., a potential host, a challenged plant, or other environmental stimulus. In terms of reproduction, having a species-specific signal in response to host cues would increase EPN reproductive success since mating occurs inside the host insect. However, this signal may be recognized by closely related or reproductively compatible species. We further hypothesize that these pheromone signals are species specific, allowing groups of the same species to aggregate, providing advantage to successfully invade and reproduce. At this stage, we may observe emergence of leaders and followers within a local population^46,47^.

Understanding how pheromone signals from host cadavers affect EPN behavior sheds light on natural insect population regulation by EPNs and has potential commercial applications. Temperatures between 15 °C and 20 °C are common under field conditions and *S. carpocapsae* IJs are quiescent for 30 min to 24 h at these temperatures. A 30 min quiescent period following application may not be a problem in some commercial applications, but 24 h is a problem because EPNs are susceptible to desiccation and UV light exposure. *S. carpocapsae* IJs may not experience the same increase in quiescence when they are stored at 4 °C in commercial settings as we observed at 20 °C, but the longer quiescence at lower field temperatures will still affect field efficacy. Pheromone extracts from host cadavers can predictably overcome this quiescence in older IJs, although physical agitation (e.g., Fig. 6) may be an effective substitute for overcoming quiescence. Since pheromones stimulate IJ dispersal^22^, pheromone extracts from host cadavers can be used to stimulate IJ dispersal leading to a higher insect encounter rate and improved EPN efficacy as shown by Oliveira-Hofman *et al*.^23^. Understanding how EPN behave in the absence and presence of pheromone signals allows us to reduce the amount of pheromone and the number of the IJs that are required for effective treatment.

## Materials and methods

### Rearing EPNs

*S. carpocapsae* (All strain) and *S. feltiae* (SN strain) IJs (ARBICO Organics, Tucson, AZ), *S. scapterisci, S. riobrave* (355)*, S. glaseri* (11a&7b strain), *S. diaprepesi*, *H. bacteriophora* (HP88 strain), *H. indica* (HOM1 strain) and *H. floridensis* (K22 strain) have been kept in culture using *Galleria mellonella* in the laboratory. To maintain cultures, commercially obtained *G. mellonella* larvae (Wax worms, Grubco, Hamilton, OH or Vanderhorst Wholesale Inc. St. Marys, OH) were exposed to 100 IJs per larva. Infected *G. mellonella* larvae were incubated for 4 days at RT (20 + 1 °C) and insect cadavers were transferred to White traps for IJ collection^1,52,53^.

### Removing pheromones and storing IJs in the absence of dispersal pheromones from consumed host cadavers

To detect a pheromone response, nematodes need to be sensitized to pheromones by removing them^22,23,30,48^. Pheromones and other metabolites were removed from IJs according to the method established by Kaplan *et al*.^22,37,48,54^. Briefly, *S. carpocapsae* or *S. feltiae* IJs were removed from the White trap after 4 days from the beginning of emergence and were rinsed 3 times in deionized water or ELGA Purelab Ultra (High Wycombe, UK) to remove residual cadaver-derived pheromones. Rinsed IJs were stored at 20 ± 1 °C until used in all experiments with a density of 24,000 IJs/ml unless otherwise stated. The two exceptions are followings; IJs in in Fig. 6 were stored at 14± 1 °C for 4 days and IJs in Fig. 5 were stored at 23± 1 °C for over 60 days.

Mechanical disturbance, like shaking, increases *S. feltiae* dispersal temporarily for 24 hours. On the other hand, *S. carpocapsae* IJ quiescent period were not affected by mechanical disturbances. To eliminate the effect of mechanical disturbance on dispersal, the IJs were placed in a 6 cm petri dish in 5 ml of water to provide a shallow water for aeration without shaking or mechanically disturbing the dish.

### Quiescent period quantification

The IJs (~200) in 10 µL of water were placed in the center of an agar plate and excess water was absorbed by the media. The quiescent period was considered to begin when the water was absorbed by the agar medium and IJs became motionless. Quiescence ended when the first IJs resumed motion and started moving away from the 1.3 cm diameter IJ placement site (Fig. 3A).

### Dispersal assays and quantification

Dispersal assays were conducted as described by Kaplan *et al*.^22^ with two modifications to the assay run time. Briefly, residual dispersal pheromones were removed from the IJs by rinsing them in deionized water 3 times, and the IJs were then stored in deionized water at 20 °C in all experiments unless stated otherwise. On day 0 (the day pheromone was removed) and at 2 day intervals thereafter, ~200–300 IJs of either species in 10 µL of water were placed in the center of an agar plate (Fig. 3A) with 6 cm diameter Petri dishes, waited until the water was absorbed by the media and IJs were free to move from the deployment site. The assays were conducted at the same time of the day in the mornings. The experiment was stopped by collecting IJs after 30 minutes; IJs remaining inside the 1.3 cm ring were considered non-dispersed (Fig. 3A), and those that left the placement ring were considered to have dispersed. If there was a quiescent period, IJs were allowed to disperse for 30 min after the quiescent period ended. Agar was at 0.9% with a gel strength >/= 900 g/cm^2^ (Caisson Agar, Type I, Smithfield, UT).

### Preparation of pheromone extract

Pheromones were extracted from *S. carpocapsae, S. feltiae*, *S. scapterisci, S. riobrave, S. glaseri*, *S. diaprepesi*, *H. bacteriophora*, *H. indica* and *H. floridensis* infected and consumed *G. mellonella* grubs in 70% methanol as by Kaplan *et al*.^22^. Infected cadavers were harvested within 10 days of IJ emergence. Then the cadavers were mixed with 70% methanol (one cadaver in 1 mL of 70% methanol) in an incubator shaker (New Brunswick Scientific,) with a speed of 150 rpm shaker at RT for 10 minutes. The supernatant was collected by centrifugation at 5000 g for 15 min and dried in a rotary evaporator. The extract was then resuspended in 10X concentration using purified water (ELGA Purelab Ultra, High Wycombe, UK) and centrifuged at 6,000 g for 15 min. The supernatant was lyophilized in a Labconco Freeze Dryer (Labconco floor model Casscade FreeZone 12 L, Kansas City, MO) and stored at −80 °C.

### Pheromone treatment

A physiologically relevant concentration of pheromone extract was used for the pheromone treatment as described in Kaplan *et al*.^22,23^. Briefly, each *G. mellonella* were considered as 200 µL and extracts from consumed waxworms were resuspended in water to prepare 10X stock. When 10 µL water containing suspended *S. carpocapsae* or *S. feltiae* IJs was placed on the agar, a 1 µL aliquot of 10X pheromone extracts was added to the suspension. It took 10–15 min for the media to absorb the liquid and for the IJs to thus be able to move. IJs were allowed to disperse for 30 min for quantification.

### Experiments at temperatures from 15 °C to 30 °C for dispersal and quiescent period

#### *S. feltiae* 12-day time course experiment

*S. feltiae* IJ dispersal was observed every other day during a 12-day period starting on day 0 and ending on day 12 or 14 at room temperature (RT), 20 ± 1 °C, in the absence of dispersal pheromones from host cadavers (Fig. 3B). Assays were conducted twice with separate culture batches of nematodes (presented on Fig. 3B and Supplemental Table S2), and started on different days with 3 or 4 replicate plates per treatment per time point per run; a total of 53 plates were assessed. Statistical differences were analyzed using linear regression model (R Studio with R version 6.3.1). R^2^ is 0.93 for *S. feltiae* dispersal.

#### *S. carpocapsae* temperature experiments

*S. carpocapsae* IJ quiescent period and dispersal were measured (Fig. 4A,C, Supplemental Fig. S1, Table S5) twice, once for a period of 60 days (0, 4, 21, 32, 46, 60 days), and once for 30 days (0, 1, 4, 10, 18, 30 days) at temperatures from 30 °C to 15 °C in 5 °C increments. *S. carpocapsae* IJ quiescent period was determined a third time for 12 days (0, 2, 4, 6, 8, 10, 12) at temperatures from 30 °C to 15 °C in 5 °C increments using a different methodology (Supplemental Table S4). The IJs storage temperature was at 20 ± 1 °C for all the experiments unless otherwise stated. Agar plates were conditioned to test temperatures prior to assay as 15, 20, 25, 30 °C. IJs were placed in 10 µl of water onto temperature conditioned agar plates and then into incubators with respective temperatures. Quantification of the quiescent period was followed by 30 min dispersal. Only the 15 °C and 20 °C temperature treatment were set up with a paired pheromone treatment (Fig. 4B,D and Supplemental Table S5) because we were testing stimulation of dispersal during the quiescent period and IJs at 25 and 30 °C did not show a quiescent period to test.

For temperatures 25 and 30 °C, since there was no quiescence for high temperatures, their dispersal was quantified and presented in Supplemental Fig. S1A and Table S5. For temperatures 15 and 20 °C in Fig. 4A,C and Supplemental Tables S4 and S5, 248 agar plates were analyzed in the presence and absence of pheromone extracts. Because populations of IJs with no pheromone extracts (96 plates) showed quiescence and in a side by side experiment, populations of IJs treated with pheromone extracts (96 plates) showed no quiescent period and dispersed, the IJ dispersal reported in Fig. 4B and 4D was analyzed with linear regression; R^2^ of 0.95 and 0.64, respectively. The assays for 60-day, 30-day and 12-day temperature experiments were conducted using different batches of nematodes at different times. Assays were conducted for 60-days (presented in Fig. 4A–D and Supplemental Fig. S1), with 4 replicate plates per treatment per run; a total of 144 plates were assessed. Assays were conducted for 30-days (Supplemental Table S5), with 2 (only for day 30 of 25 °C and 30 °C treatments) or 4 replicate plates per treatment per run (total of 116 plates) and for 12-days (Supplemental Table S4) with 4 replicate plates per treatment per run (total of 112). The sum of 3-time course experiments at 4 different temperatures with and without pheromones resulted in the analysis of 372 plates.

#### *S. feltiae* experiment in the absence and presence of pheromones

*S. feltiae* IJ dispersal (Fig. 5) was determined at 20 ± 1 °C for a period of 60 days; 11, 17, 24, 45 and 60 days after removal of residual pheromones from host cadavers. The IJs were reared and stored at 23 ± 1 °C. The assays were scored qualitatively as dispersed and non-dispersed after 30 min. The day 60 data collection method was different where we used a pre-post research design. The control was water treatment since the solvent for the cadaver extracts is water. Replications (n) are presented in Fig. 5 and a total of 35 plates were analyzed.

### Crawling assays with agar with sand for testing pheromone extracts’ effect on *S. carpocapsae* ambushing behaviors

Assays of crawling prevalence were conducted on 90 mm Petri plates containing 30 mL of 2% water agar. Trials were conducted either on Petri plates without sand (agar only) or on plates that had been surface sprinkled with 0.5 g of air-dry sand (sand + agar)^25,42^. The sand used was commercial play sand passed through a 35-mesh sieve (500 micron opening) in the laboratory.

Deconditioned IJs (see the pheromone removal section above) that had been stored in tissue culture flasks at 14 °C for 4 days were suspended in 500 µL of distilled water and then exposed either to pheromone (50 µL of 10X *S*. *carpocapsae* dispersal pheromone extracts) or to 50 µL of DI water and then shaken regularly for a minimum of 20 minutes. Individual 550 µL IJ suspensions were then filtered through a 25 mm diameter P8 filter paper disc (Fisher Scientific, Pittsburgh, PA) by vacuum. IJs were transferred from the filter paper to the Petri plates using a laboratory probe.

Behavioral assessments began 10 min after IJs were transferred to the plate. The assay temperature was 22 °C. IJs were recorded as crawling or not crawling (which included jumping [attached to the lid within the field of view], waving, or tail standing). Once 100 worms had been counted, another field of view within the same plate was selected and assessed in a similar fashion. Assessments were made of three separate groups of 100 IJs per plate (total of 300 worms assessed per plate). Assays were conducted twice, with either 2 (in one case) or 3 replicate plates per treatment (factorial combination of +/− pheromone, +/− sand) per run; a total of 23 plates were assessed.

### Dispersal plate assays with agar and sand + agar for testing pheromone extracts’ effect on *S. carpocapsae*

Dispersal assays were conducted on 90 mm Petri plate arenas with 30 mL of 2% water agar. Trials were conducted either in arenas without sand (agar only) or on plates that had been surface sprinkled with 0.5 g of air-dry sieved commercial play sand (sand + agar), as described above. Deconditioned IJs (IJs kept in the absence of pheromone in water at 14 °C for 4 days) were treated as described above. Individual 550 µL IJ suspensions were then filtered through a 25 mm diameter P8 filter paper disc (Fisher Scientific, Pittsburgh, PA) by vacuum. IJs were applied to the plate by gently pressing the filter paper disc on the center of the agar surface. The disc was then removed, leaving the IJs behind; if a dish was to receive sand, the sand was then sprinkled over the agar surface. The assay temperature was 22 °C.

*S. carpocapsae* IJs were allowed to disperse across the surface of the 90 mm arena for 30 minutes; at the conclusion of the test, 30 mm and 59 mm diameter stainless steel rings were used to separate the inner, middle, and outer “rings” of the arena. IJs were washed from each of the separate rings, as well as from the lid of the arena, and counted under a stereomicroscope. A dispersal index was determined for each arena, using the number of IJs found in the outermost ring (“Outer”), the number found in the middle ring (“Middle”) and the total number of IJs recovered from the surface of the arena (“Total” – note that while this number includes Inner, Middle, and Outer IJs, it excludes IJs found on the lid, see *statistical analysis* section below). The formula used to calculate the Dispersal Index was ((59*Outer) + (30*Middle))/Total. Assays were conducted twice, with 3 replicate plates per treatment (factorial combination of +/− pheromone, +/− sand) per run; a total of 24 plates were assessed.

### Cross species test to determine whether *S. carpocapsae* IJs respond to pheromones from infected host cadavers of other EPN species

*S. carpocapsae* IJs were rinsed 3 times and tested for quiescence using the dispersal assay described in “dispersal assays and quantification” described above when exposed to physiologically relevant concentration of dispersal pheromone extracts from consumed host cadavers of *S. carpocapsae* (positive control)*, S. feltiae*, *S. scapterisci, S. riobrave, S. glaseri*, *S. diaprepesi*, *H. bacteriophora*, *H. indica, H. floridensis*, and water (negative control) on day 0. Assays were conducted twice, with either 4 or 5 replicate plates per treatment per run; a total of 90 plates were assessed in Fig. 7A. Assays conducted at 21.5 ± 1 °C. Since pheromone treated IJs did not show a quiescent period, they were allowed to disperse 30 min. Five replicates for each treatment were quantified for dispersal (a total of 50 plates) in Fig. 7B. ANOVA and Tukeys HSD test was done for pairwise comparisons (Supplemental Table 8A–C).

### Statistical analysis

Linear regression, ANOVA, Tukey’s Honestly Significant Difference (HSD), or Students’ t-tests were used to determine statistical differences using R version 3.6.1 (or 3.5.1) run in R Studio. Details of statistical analysis are in the Supplemental Methods.

## Supplementary information

Supplementary information.
