# Supplementary material for: Conspecific and heterospecific pheromones stimulate dispersal of entomopathogenic nematodes during quiescence
Source: Sci Rep. 2020 Mar 31;10:5738. doi: 10.1038/s41598-020-62817-y (PMC7109026; doi:10.1038/s41598-020-62817-y)
Supplement: Supplementary file 1 — Supplementary information. [file 41598_2020_62817_MOESM1_ESM.pdf]

Supplementary Information

Conspecific and heterospecific pheromones stimulate dispersal of entomopathogenic nematodes during quiescence

Fatma Kaplan[1*], Abigail Perret-Gentil[1,5], Julie Giurintano[1,6], Glen Stevens[2], Hilal Erdogan[2,4], Karl C. Schiller[1], Amaleah Mirti[1,7], Edith Sampson[1,7], Cedric Torres[1,8], Jiayi Sun[1,9], Edwin E. Lewis[2], David Shapiro-Ilan[3]

[1]Pheronym, Inc. Davis, CA, 95618, USA
[2]University of Idaho, Department of Entomology, Plant pathology and Nematology, Moscow, ID 83844, USA
[3]USDA-ARS, Southeastern Fruit and Tree Nut Research Laboratory, Byron, GA, 31008, USA
[4]Faculty of Agriculture, Department of Biosystems Engineering, Bursa Uludağ University, Bursa, 16059 Turkey
[5]Current address: GRACE Market Place, Gainesville, FL, 32609, USA
[6]Current address: Lewis Katz School of Medicine, Philadelphia, PA, 19140, USA
[7]Current address: University of Florida, Gainesville, FL, 32610, USA
[8]Current address: Mérieux NutriSciences, Gainesville, FL, 32641, USA
[9]Current address: Captozyme, LLC., Gainesville, FL, 32653, USA

*fkaplan@pheronym.com

Supplementary Figures

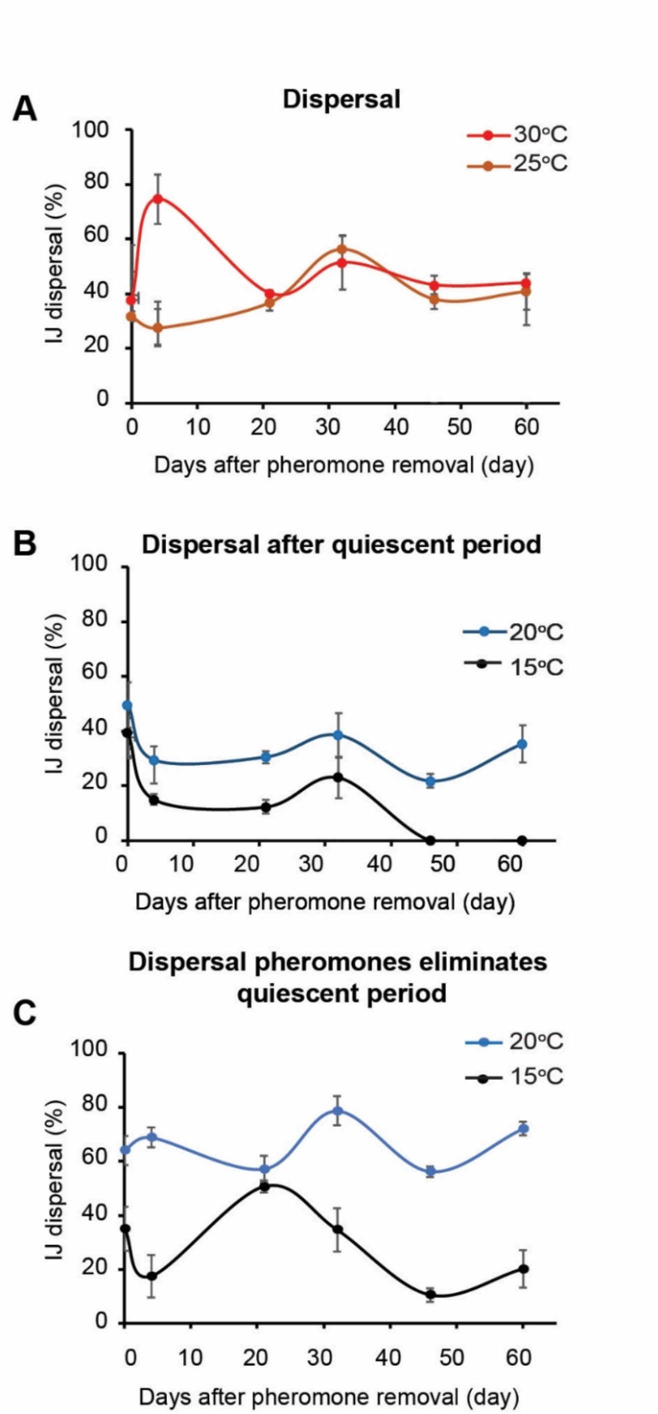

Supplementary Figure S1. *Steinernema carpocapsae* IJ population dispersal. A) *S. carpocapsae* IJ dispersal for 30 min at 25 and 30°C. B) After the quiescent period, IJs dispersal for 30 min at 20 and 15°C. No dispersal data collected at 15C for day 45 and 60 because the samples did not disperse after 24h. C) Stimulated dispersal for 30 min during the quiescent period by the dispersal pheromone extract at 20 and 15°C (the data comes from Figure 4B and D in the manuscript). The mean ± s.e.m. of 4 replications is presented.

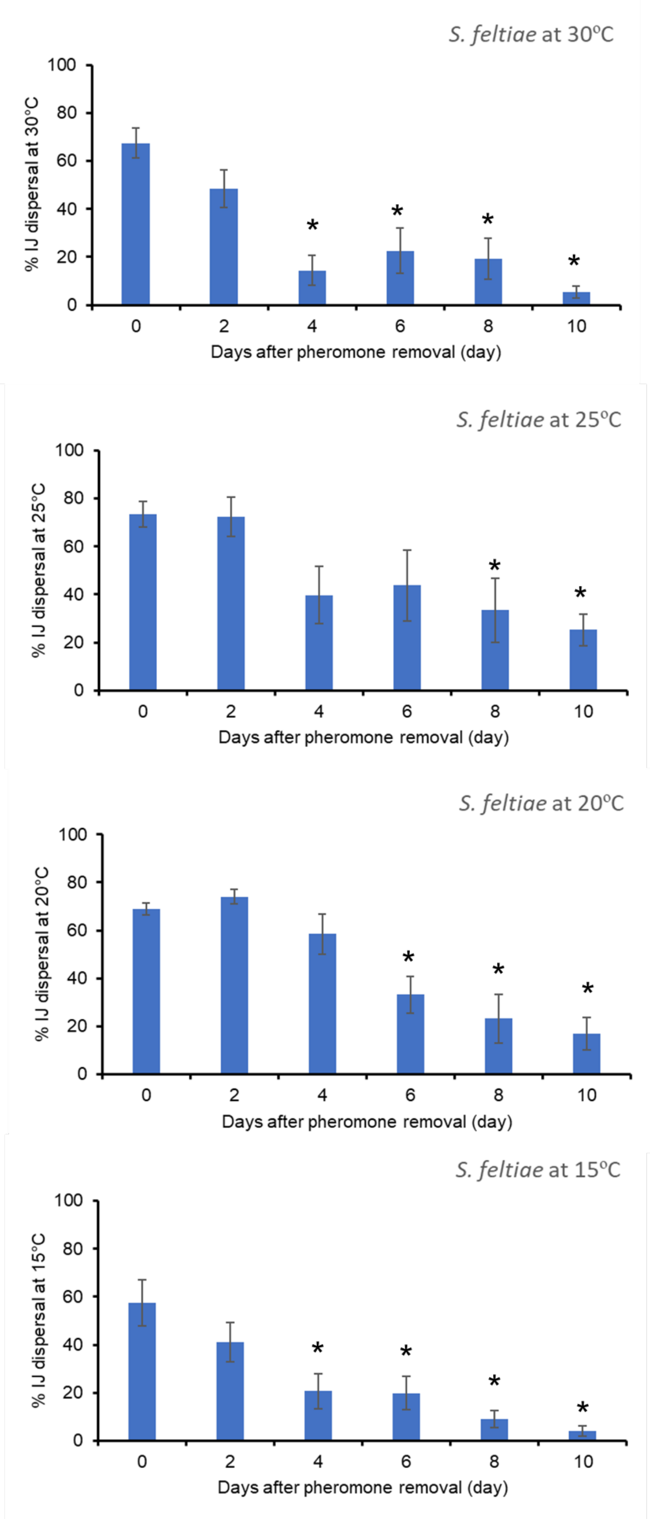

Supplementary Figure S2. A 10-day time course of *Steinernema feltiae* IJ dispersal in the absence of dispersal pheromones from consumed host cadaver extracts at 4 different temperatures from 30°C to 15°C. The mean ± s.e.m. of 8 replications from two assays is presented. "*" indicates significant differences from day 0 according to Tukey's HSD (α = 0.05)

Supplementary Tables

Supplementary Table S1. *Steinernema feltiae* IJ population percent dispersal in Fig 3B.

	Beta	P value	
**Intercept**	**83.147**	**2.14E-10**	***
**Day 2**	**-59.583**	**1.16E-06**	***
**Day 4**	**-53.61**	**3.91E-06**	***
**Day 6**	**-76.04**	**6.06E-08**	***
**Day 8**	**-82.967**	**2.02E-08**	***
**Day 10**	**-80.838**	**2.81E-08**	***
**Day 12**	**-78.512**	**4.06E-08**	***

Residual standard error: 8.994 on 14 degrees of freedom
Multiple R-squared: 0.9321, Adjusted R-squared: 0.903
F-statistic: 32.04 on 6 and 14 DF, p-value: 2.116e-07

Supplemental Table 2. Dispersal of *S. feltiae* IJs in the absence of pheromone extracts from consumed host cadavers at 20°C

Days after rinsing (day)	n	Dispersal (%+sem)
0	4	87+3
2	4	49+7
4	4	20+6
6	4	9+6
8	4	20+7
10	4	21+8
12	4	16+7
14	4	21+7

Supplementary Table S3. Regression analysis of *S. feltiae* IJ population (data is from Supplemental Table S2) percent dispersal at 20°C.

	Beta	P-value	
**Intercept**	**86.712**	**1.3E-12**	***
**Day 2**	**-38.002**	**0.000368**	***
**Day 4**	**-66.957**	**0.000000154**	***
**Day 6**	**-78.052**	**1.05E-08**	***
**Day 8**	**-66.364**	**0.000000179**	***
**Day 10**	**-66.086**	**0.000000192**	***
**Day 12**	**-70.705**	**6.09E-08**	***
**Day 14**	**-65.806**	**0.000000206**	***

Residual standard error: 12.98 on 24 degrees of freedom
Multiple R-squared: 0.8195, Adjusted R-squared: 0.7668
F-statistic: 15.56 on 7 and 24 DF, p-value: 1.599e-07

Supplemental Table S4. The 12-day time course quiescence of *S. carpocpasae* IJs in the absence of pheromone extracts from consumed host cadavers at 4 different temperatures.

Temperature ºC $\pm$1	Days after rinsing (day)		Pheromone Extract	Plates IJs had quiescent period
**30ºC Time course without Pheromone extracts**				
	0		No	0/4
	2		No	0/4
	4		No	0/4
	6		No	0/4
	8		No	0/4
	10		No	0/4
	12		No	0/4
**25ºC Time course without Pheromone extracts**				
	0		No	0/4
	2		No	0/4
	4		No	0/4
	6		No	0/4
	8		No	0/4
	10		No	0/4
	12		No	0/4
**20ºC Time course without Pheromone extracts**				
	0		No	4/4
	2		No	4/4
	4		No	4/4
	6		No	4/4
	8		No	4/4
	10		No	4/4
	12		No	4/4
**15ºC Time course without Pheromone extracts**				
	0		No	4/4
	2		No	4/4
	4		No	4/4
	6		No	4/4
	8		No	4/4
	10		No	4/4
	12		No	4/4

Supplemental Table S5. The 30-day time course quiescence and dispersal of *S. carpocpasae* IJs in the absence and presence of pheromone extracts from consumed host cadavers.

Temperature ºC ($\pm$1)	Days after rinsing (day)	n	Pheromone Extract	Quiescence (min $\pm$sem)	30 min Dispersal (% +sem)	30 min Dispersal After quiescence (% +sem)	Total assay time (Quiescence + 30 min dispersal) (min)
**30ºC Time course without Pheromone extracts**							
	0	4	No	0	82 $\pm$7		30 (0+30)
	4	4	No	0	76 $\pm$6		30 (0+30)
	30	2	No	0	Dispersed		30 (0+30)
**25ºC Time course without Pheromone extracts**							
	0	4	No	0	82 $\pm$5		30 (0+30)
	4	4	No	0	64 $\pm$4		30 (0+30)
	30	2	No	0	Dispersed		30 (0+30)
**20ºC Time course without Pheromone extracts**							
	0	4	No	44 $\pm$8		76 $\pm$3	74 (44+30)
	1	4	No	53 $\pm$3		71 $\pm$5	83 (53+30)
	4	4	No	88 $\pm$3		44 $\pm$3	118 (88+30)
	10	4	No	110 $\pm$4		55 $\pm$9	140 (110+30)
**	18	4	No	124 $\pm$4		73 $\pm$6	154 (124+30)
*	30	4	No	95 $\pm$26		30 $\pm$8	125 (95+30)
**20°C Time course with Pheromone extracts**							
	0	4	Yes	0	70 +4		30 (0+30)
	1	4	Yes	0	75 $\pm$4		30 (0+30)
	4	4	Yes	0	78 $\pm$8		30 (0+30)
	10	4	Yes	0	70 $\pm$4		30 (0+30)
**	18	4	Yes	0	89 $\pm$1		30 (0+30)
*	30	4	Yes	0	85 $\pm$3		30 (0+30)
**15ºC Time course without Pheromone extracts**							
	0	4	No	113 $\pm$6		41 $\pm$6	143 (113+30)
	1	4	No	126 $\pm$5		48 $\pm$4	156 (126+30)
	4	4	No	142 $\pm$1		18 $\pm$1	172 (142+30)
	10	4	No	128 $\pm$6		36 $\pm$5	158 (128+30)
	18	4	No	146 $\pm$3		59 $\pm$4	176 (146+30)
	30	4	No	150 $\pm$4		10 $\pm$2	180 (150+30)
**15°C Time course with Pheromone extracts**							
	0	4	Yes	0	56 $\pm$5		30 (0+30)
	1	4	Yes	0	45 $\pm$7		30 (0+30)
	4	4	Yes	0	33 +1		30 (0+30)
	10	4	Yes	0	22 $\pm$5		30 (0+30)
	18	4	Yes	0	53 +2		30 (0+30)
	30	4	Yes	0	46 $\pm$5		30 (0+30)

*Temperature went up temporarily to 22.5°C **Temperature temporarily went up to 21°C

Supplementary Table S6. Regression analysis of *S. carpocapsae* IJ population dispersal at 20°C in Fig 4B.

	Estimate	Std.	T value	t value
(Intercept)	-0.46508	2.10104	-0.221	0.826
**Pheromone**	**66.81082**	**2.21491**	**30.164**	**<2e-16**
Days	0.01712	0.05155	0.332	0.741

Residual standard error: 7.673 on 45 degrees of freedom
Multiple R-squared: 0.9529, Adjusted R-squared: 0.9508
F-statistic: 455 on 2 and 45 DF, p-value: < 2.2e-16

Supplementary Table S7. Regression analysis for *S. carpocapsae* IJ population dispersal at 15°C in Fig 4D.

	Estimate	Standard error	T value	Pr(>|t|)
(Intercept)	3.11422	2.85597	1.09	0.281
**Pheromone**	**27.91304**	**3.01075**	**9.271**	**5.27E-12**
Days	-0.11463	0.07008	-1.636	0.109

Residual standard error: 10.43 on 45 degrees of freedom
Multiple R-squared: 0.6632, Adjusted R-squared: 0.6483
F-statistic: 44.31 on 2 and 45 DF, p-value: 2.315e-11

Supplementary Table S8A. Pairwise comparisons; negative control (water) vs dispersal pheromone extracts from multiple species using *S. carpocapsae* IJ for the data in Figure 7B.

Extract Species	Difference	Lower bound	Upper bound	Adjusted p-value	
H. bacteriophora	**32.9524899**	**9.045112**	**56.859868**	**0.0014851**	*
H. floridensis	**74.1358654**	**50.228487**	**98.043244**	**0**	*
H. indica	**81.8644748**	**57.957097**	**105.771853**	**0**	*
S. carpocapsae	**81.2876022**	**57.380224**	**105.19498**	**0**	*
S. diaprepesi	**54.0841192**	**30.176741**	**77.991497**	**0.0000001**	*
S. feltiae	**49.0603938**	**25.153016**	**72.967772**	**0.0000012**	*
S. glaseri	**65.5085715**	**41.601193**	**89.41595**	**0**	*
S. riobrave	**59.6664628**	**35.759085**	**83.573841**	**0**	*
S. scapterisci	**74.7688983**	**50.86152**	**98.676276**	**0**	*

*shows statistically significant response

Supplementary Table S8B. Pairwise comparisons; positive control (*S. carpocapsae* dispersal pheromone extracts) vs dispersal pheromone extracts from multiple species using *S. carpocapsae* IJ for the data in Figure 7B.

Extract Species	Difference	Lower bound	Upper bound	Adjusted p-value	
H. bacteriophora	**48.3351123**	**72.242491**	**24.427734**	**0.0000017**	*
H. floridensis	7.1517368	16.755641	31.059115	0.9905053	
H. indica	0.5768726	23.330506	24.484251	1	
S. diaprepesi	**27.203483**	**51.110861**	**3.296105**	**0.0151027**	*
S. feltiae	**32.2272084**	**56.134587**	**8.31983**	**0.0020138**	*
S. glaseri	15.7790307	39.686409	8.128347	0.4672204	
S. riobrave	21.6211394	45.528518	2.286239	0.1057008	
S. scapterisci	6.5187039	30.426082	17.388674	0.9951261	

*shows statistically significant response

Supplementary Table S8C. ANOVA of *S. carpocapsae* dispersal in response to other species' dispersal pheromone extracts in Figure 7B.

	Degrees of freedom	Sum of squares	Mean square	F value	P value
Pheromone Extracts	9	28974	32.19	25.25	8.27E-14
Residuals	40	5100	127		

Supplemental Table S9A. Regression analysis of S. feltiae IJ population percent dispersal at 30°C

Day	Beta	P-value	
Day0	67.445	6.72E-13	***
Day2	-10.893	0.252	
Day4	-51.284	2.29E-06	***
Day6	-44.596	2.34E-05	***
Day8	-48.774	5.52E-06	***
Day10	-61.414	6.43E-08	***

Residual standard error: 18.75 on 42 degrees of freedom
Multiple R-squared: 0.6224, Adjusted R-squared: 0.5774
F-statistic: 13.84 on 5 and 42 DF, p-value: 5.306e-08
Signif. codes: 0 ‘***’ 0.001 ‘**’ 0.01 ‘*’ 0.05 ‘.’ 0.1 ‘ ’ 1

Supplemental Table S9B. Regression analysis of S. feltiae IJ population percent dispersal at 25°C

Day	Beta	P-value	
Day0	68.009	3.76E-08	***
Day2	3.034	0.83253	
Day4	-24.65	0.09131	.
Day6	-20.862	0.16497	
Day8	-34.611	0.01966	*
Day10	-42.816	0.00453	**

Residual standard error: 28.51 on 41 degrees of freedom
(1 observation deleted due to missingness)
Multiple R-squared: 0.2882, Adjusted R-squared: 0.2014
F-statistic: 3.32 on 5 and 41 DF, p-value: 0.01311
Signif. codes: 0 ‘***’ 0.001 ‘**’ 0.01 ‘*’ 0.05 ‘.’ 0.1 ‘ ’ 1

Supplemental Table S9C. Regression analysis of S. feltiae IJ population percent dispersal at 20°C

Day	Beta	P-value	
Day0	60.224	1.28E-10	***
Day2	12.067	0.234033	
Day4	4.485	0.666806	
Day6	-22.225	0.03168	*
Day8	-37.024	0.000624	***
Day10	-43.41	8.95E-05	***

Residual standard error: 19.98 on 41 degrees of freedom
(1 observation deleted due to missingness)
Multiple R-squared: 0.5628, Adjusted R-squared: 0.5094
F-statistic: 10.55 on 5 and 41 DF, p-value: 1.465e-06
Signif. codes: 0 ‘***’ 0.001 ‘**’ 0.01 ‘*’ 0.05 ‘.’ 0.1 ‘ ’ 1

Supplemental Table S9D. Regression analysis of S. feltiae IJ population percent dispersal at 15°C

Day	Beta	P-value	
Day0	49.499	4.26E-09	***
Day2	-1.195	0.899849	
Day4	-29.874	0.002916	**
Day6	-28.776	0.005288	**
Day8	-39.324	0.000155	***
Day10	-45.459	2.02E-05	***

Residual standard error: 18.87 on 41 degrees of freedom
(1 observation deleted due to missingness)
Multiple R-squared: 0.5028, Adjusted R-squared: 0.4421
F-statistic: 8.291 on 5 and 41 DF, p-value: 1.753e-05
Signif. codes: 0 ‘***’ 0.001 ‘**’ 0.01 ‘*’ 0.05 ‘.’ 0.1 ‘ ’ 1

Supplementary Methods

Statistical analysis

Data analysis in Fig 3 and Fig 4: Linear regression model was used to analyze the data in Fig 3B, Fig 4B and D and Supplemental Table 2. for the details of analysis see Supplementary Table 1 for Fig. 3B, Supplemental Table S3 for 14-day time course data in Supp. Table S2 and Supplementary Table S6 (for Fig. 4B) and Supplementary Table S7 (for Fig 4D). Analyses were conducted using R version 3.6.1 run in R Studio.
Data analysis in Fig 6: Changes at $p \leq 0.05$ were considered significant. "*" indicates $p<0.05$. Analysis of crawling prevalence assays was conducted using ANOVA. We assessed whether the addition of sand and/or pheromone influenced the percentage of crawling nematodes. In Fig 6., dispersal plate assays were also analyzed using ANOVA. We assessed whether the presence of sand and/or pheromone influenced the number of IJs found on the dish lids (i.e., jumping rates) and dispersal rates (mm moved per IJ over the 30-minute assay period). Nematodes that were found on the lid were excluded from the dispersal assessment, as there was no way to know from which point on the dish, they had initiated their jump. Post-hoc analyses were conducted using Tukey's Honestly Significant Difference (HSD) test. Analyses were conducted using R version 3.5.1 run in RStudio. Data analysis in Fig 7 and Supplemental Fig. S2: ANOVA and Tukeys HSD test was done for pairwise comparisons (Supplementary Table 8A, B and C and Supplementary Table S9A, B, C, and D). Analyses were conducted using R version 3.6.1 run in R Studio.

S. feltiae and *S. carpocapsae* rearing, storage conditions, density and dispersal assays

The methods for rearing, storage conditions, density, quantification of quiescence and dispersal assays were described in the manuscript unless otherwise stated.

S. carpocapsae 60-day time course experiments for dispersal: *S. carpocapsae* dispersal was quantified at temperatures from 15°C to 30°C. The material and method for this experiment (Supplemental Fig. S1) is described in the M&M of manuscript under the subtitle "*Experiments at temperatures from 15°C to 30°C for dispersal and quiescent period- subtitle: S. carpocapsae temperature experiments*" because the experiment started with quiescent period followed by quantification of dispersal after quiescent period. For 25 and 30°C, 48 plates were analyzed, total.

S. feltiae temperature experiments: *S. feltiae* IJ dispersal was observed every other day during a 10-day period starting on day 0 and ending on day 10 in the absence of dispersal pheromones from host cadavers at temperatures from 15ºC to 30ºC in 5°C increments. The IJs storage temperature was at 20±1ºC for all the experiments. Agar plates were conditioned to test temperatures (15, 20, 25, 30ºC) prior to assay. IJs were placed in 10 µl of water onto temperature conditioned agar plates and then into incubators set to their respective temperatures. Assays were run for 60 min. Assays were conducted twice in time with separate culture batches of nematodes, starting on different days, with 3 or 4 replicate plates per treatment per time point per run; a total of 189 plates were assessed (Supplemental Fig. S2). For statistical analysis see Supplemental Table S9.
